# Inflammatory cell death induced by 5-aminolevulinic acid-photodynamic therapy initiates anticancer immunity

**DOI:** 10.3389/fonc.2023.1156763

**Published:** 2023-10-02

**Authors:** Lingyan Wang, Vipin Shankar Chelakkot, Nick Newhook, Stephanie Tucker, Kensuke Hirasawa

**Affiliations:** ^1^ Division of BioMedical Sciences, Faculty of Medicine, Memorial University of Newfoundland, St. John’s, NL, Canada; ^2^ Medical Laboratories, Faculty of Medicine, Memorial University of Newfoundland, St. John’s, NL, Canada

**Keywords:** 5-aminolevulinic acid, photodynamic therapy, pyroptosis, necroptosis, anticancer immunity

## Abstract

**Background:**

Inflammatory cell death is a form of programmed cell death (PCD) that induces inflammatory mediators during the process. The production of inflammatory mediators during cell death is beneficial in standard cancer therapies as it can break the immune silence in cancers and induce anticancer immunity. Photodynamic therapy (PDT) is a cancer therapy with photosensitizer molecules and light sources to destroy cancer cells, which is currently used for treating different types of cancers in clinical settings. In this study, we investigated if PDT using 5-aminolevulinic (5-ALA-PDT) causes inflammatory cell death and, subsequently, increases the immunogenicity of cancer cells.

**Methods:**

Mouse breast cancer (4T1) and human colon cancer (DLD-1) cells were treated with 5-ALA for 4 hours and then irradiated with a light source. PCD induction was measured by western blot analysis and FACS. Morphological changes were determined by transmission electron microscopy (TEM). BALB/c mice were injected with cell-free media, supernatant of freeze/thaw cells or supernatant of PDT cells intramuscular every week for 4 weeks and then challenged with 4T1 cells at the right hind flank of BALB/c. Tumor growth was monitored for 12 days.

**Results:**

We found that 5-ALA-PDT induces inflammatory cell death, but not apoptosis, in 4T1 cells and DLD-1 cells *in vitro*. Moreover, when mice were pretreated with 5-ALA-PDT culture supernatant, the growth of 4T1 tumors was significantly suppressed compared to those pretreated with freeze and thaw (F/T) 4T1 culture supernatant.

**Conclusion:**

These results indicate that 5-ALA-PDT induces inflammatory cell death which promotes anticancer immunity *in vivo*.

## Introduction

Photodynamic therapy (PDT) is a minimally invasive treatment to destroy cancer cells by combining treatment with photosensitizers (PSs) and light exposure at an appropriate wavelength (1). PSs accumulated in cancer cells absorb light energy and are excited from a ground singlets state to an excited singlet state, followed by a transition to a relatively long-lived excited triplet state (2). At the excited triplet state, the PSs initiate type I and type II photosensitized oxidation reactions. In the type I reaction, PSs transfer electrons to neighbouring biomolecules to produce reactive oxygen species (ROS). Moreover, type II reactions generate singlet oxygen by direct interaction of PSs with molecular oxygen which produces a variety of ROS. The production of ROS plays a primary role in causing cancer cell death induced by PDT (3).

5-aminolevulinic acid (5-ALA) is a natural amino acid which can lead to the generation of protoporphyrin IX (PpIX) via the heme biosynthesis pathway (4). PpIX is further converted into heme in the mitochondria by ferrochelatase (FECH) (5). As the heme biosynthesis pathway is highly activated in transformed cells, PpIX, which is a photosensitizer, can be accumulated in most cancer cells more efficiently than in normal cells when treated with exogenous 5-ALA (6). Accumulated PpIX in cancer cells can be utilized during PDT to induce ROS when excited with light at the specific wavelength, leading to cancer cell death. PDT using 5-ALA (5-ALA-PDT) has been established as a promising alternative to conventional cancer therapies for treating different types of cancers such as bladder, brain, colon, head and neck, lung, oesophagus, oral and skin (7–11). Additionally, PpIX is an endogenous fluorophore that exhibits red fluorescence, which can be used for intraoperative visualization of tumors known as photodynamic diagnosis (PDD) (12). PDD using 5-ALA (5-ALA-PDD) is recently approved for an optical imaging method for Grade III and IV gliomas during surgery by the FDA (13).

**Figure 1:**
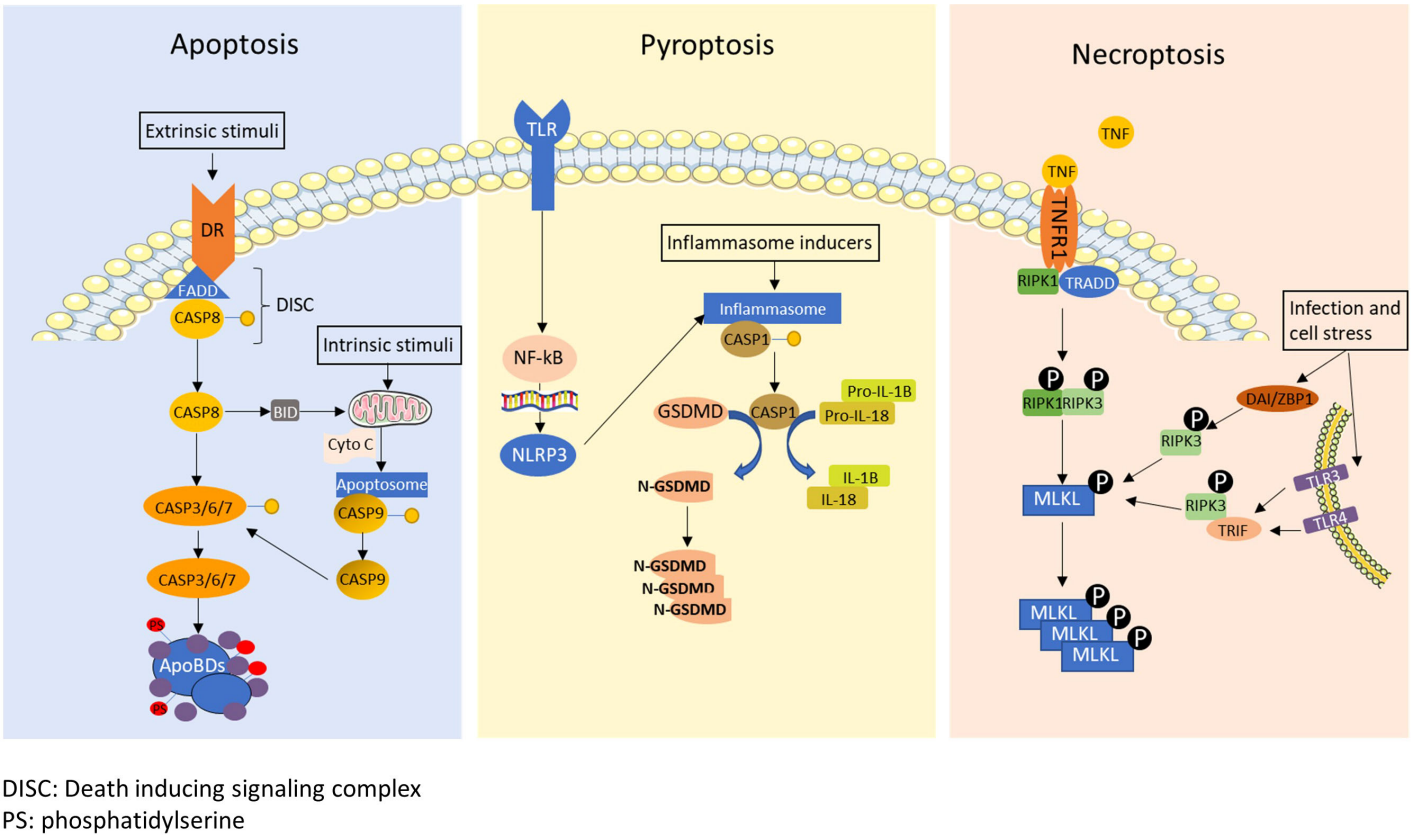
A schematic diagram of apoptosis, pyroptosis and necroptosis. Apoptosis: The death receptors recruit Fas-associate death domain (FADD) and caspase-8 to form death-inducing signaling complex (DISC) under extrinsic stimuli. The DISC cleaves caspase-8, and then cleaved caspase-8 initiates the formation of apoptotic bodies by activating caspase-3, 6 and 7. Caspase-8 also cleaves BH3 interacting-domain death agonist (BID), which translocate to mitochondria to induce cytochrome C (Cyto C) release. Internal stimuli such as cell damage, DNA damage, cellular stresses, and irradiation also trigger release of cytochrome C as the intrinsic pathway. Released cytochrome c forms apoptosome which cleaves caspase-9. Cleaved caspase-9 further cleaves and activates caspase-3, 6 and 7. Pyroptosis: Inflammatory signals or infections trigger pyroptosis by activating inflammasome formation. Activated inflammasome cleaves and activates caspase-1 and subsequently gasdermin D (GSDMD). Cleaved GSDMD translocates to the membrane and causes membrane rupture. Activated caspase-1 also cleave pro-IL1β and pro-IL-18 into mature IL-1β and IL-18. Necroptosis: Necroptosis is typically induced by tumor necrosis factor alpha (TNF-α), infection or cellular stress. TNF-α signal induces phosphorylation of receptor-interacting serine/threonine-protein kinase 1 (RIPK1) and 3 (RIPK3), resulting in the subsequent phosphorylation of mixed lineage kinase domain like pseudokinase (MLKL). Moreover, MLKL can be phosphorylated through Z-DNA-binding protein 1 (ZBP1)/RIPK3, Toll-like receptors 3 (TLR3)/TIR-domain-containing adapter-inducing interferon-β (TRIF)/RIPK3 or TLR4/TRIF/RIPK3 pathway upon cellular stress and microbial or viral infection.

Most cancer cells die by activation of programmed cell death (PCD) mechanisms during cancer treatments (**Figure 1**) (14, 15). Apoptosis, the first identified PCD, is characterized by chromatin condensation and DNA fragmentation, which is considered as non-immunogenic cell death (16, 17). Apoptosis can be triggered by the stimulation of death receptors (extrinsic pathway) or by various internal or external stimulation such as cell damage, DNA damage, cellular stresses, and irradiation (intrinsic pathway). Upon activation of the extrinsic pathway by binding of death ligands with death receptors, Fas-associate death domain (FADD) and caspase-8 form a death-inducing signaling complex to cleave caspase-8 (18). Activation of the intrinsic pathway leads to the release of cytochrome C in the mitochondria, which induces the apoptosome to cleave caspase-9 (19). Both activated caspases-8 and 9 cleave caspase-3, 6 and 7, and subsequently causing the formation of apoptotic bodies that lead to cell death (20). Calreticulin (CRT), which is known as another apoptosis marker, is translocated from the endoplasmic reticulum (ER) to the cellular membrane when cells undergo apoptosis, which stimulates phagocytosis (21). On the other hand, pyroptosis and necroptosis are classified as inflammatory cell death that produce cytokines and inflammatory mediators during their PCD process (22, 23). For induction of pyroptosis, danger signals derived from host cells or intracellular pathogens induce the formation of the inflammasome, which cleaves and activates caspase-1 (24). Activated caspase-1 further cleaves gasdermine D, which translocates to the cell membrane and forms pores to release intracellular contents and induce membrane rupture (25). Activated caspase-1 also cleaves the precursors of interleukin-1β (IL-1β) and IL-18 into their active forms, which are released during pyroptosis (26). Necroptosis is defined as a programmed form of necrosis. Necroptosis can be induced by a variety of stimulation such as activation of Toll-like receptor (TLR), tumor necrosis factor (TNF), cellular stresses, and microbial or viral infection (27). As a classic necroptosis inducer, TNF binds with the TNF receptor, which recruits the receptor interacting protein kinases (RIPK1) and TNFRSF1A associated via death domain (TRADD), inducing the phosphorylation of RIPK1 and RIPK3 (28). The phosphorylated complex of RIPK1 and RIPK3 phosphorylates MLKL (29). Alternatively, microbial or viral infection and cellular stresses that phosphorylates RIPK3 through Z-DNA binding protein 1(ZBP1) (30) or TIR-domain-containing adapter-inducing interferon-β (TRIF) (31). Phosphorylated MLKL translocates to the membrane and causes membrane rupture. Phosphorylated RIPK3 is also capable of inducing MLKL phosphorylation (32). Cells undergoing necroptosis release inflammatory cytokines, damage-associate molecular patterns (DAMPs), high mobility box 1 protein (HMGB1) and ATPs to promote inflammatory responses (33–35).

As cancer cells undergo multiple genetic changes, they generate mutated proteins that can be presented on their MHC class I molecules to immune cells (36). On the other hand, to avoid immune elimination, tumors evade the immune system through various mechanisms such as suppressing immune system (37), causing immune exhaustion (38), and limiting antigen presentation (39). As standard cancer therapies have been developed to directly kill cancer cells and shrink tumors, it would be beneficial for cancer survival and cure if immune evasion is broken, and anticancer immunity is induced. Cancer therapies such as chemotherapy (40), radiotherapy (41, 42) and oncolytic virus therapy (43) have been reported to activate cancer immune surveillance and anticancer immunity. To evoke anticancer immunity within tumors during cancer therapies, induction of inflammatory cell death is the crucial first step. Until now, it remains to be determined whether 5-ALA-PDT could induce inflammatory cell death and break immune silence caused by tumors though previous studies reported that 5-ALA-PDT induces apoptosis (44, 45). Here, we report that 5-ALA-PDT induces pyroptosis and necroptosis in mouse and human cancer cells, leading to the induction of anticancer immunity in an animal model of cancer.

## Methods

### Cells and reagents

Mouse 4T1 mammary gland tumor cells were obtained from Dr. Jean Marshall (Dalhousie University, Halifax, Canada). Human colon cancer DLD-1 cells were obtained from the American Type Culture Collection (ATCC; Manassas, VA, USA). 5-aminolevulinic acid (5-ALA), NLRP inhibitor (MCC950), RIPK 1 inhibitor (Nec-1s), RIPK 3 inhibitor (Dabrafenib) were purchased from Sigma (Oakville, ON). Caspase-1 inhibitor (Z-WEHD-FMK) and caspase-3 inhibitor (Z-DEVD-FMK) were purchased from R&D systems (Minneapolis, MN). Cell counting Kit (WST-8/CCK8) (ab228554) (Abcam, Boston, MA) was used to determine the cell viability. LDH assay kit (Thermo Fisher Scientific, Mississauga, ON) was used to measure amounts of the lactate dehydrogenase released into the cell culture medium to determine the plasma membrane damages.

### Cell culture

4T1 and DLD-1 cells were maintained in high glucose Dulbecco’s modified Eagle’s medium (DMEM) (Thermo Fisher Scientific), supplemented with 10% fetal bovine serum (FBS) and antibiotic-antimycotic mixture (Thermo Fisher Scientific) (100 units/mL penicillin G sodium). Cells plated in 24 or 48 well-plates were treated with 5-ALA (50 μM (4T1) or 250 μM (DLD-1)) for 4 hours and then irradiated using a Theralase TLC 3000A modular light source for 1.5 mins (Theralase Technologies Inc., Toronto, Canada; λ = 618–630 nm, fluence rate = 150 mW/cm^2^, energy density (ED) = 27 J/cm^2^). Cell viability and LDH release were measured using a CCK-8 assay kit and an LDH cytotoxicity assay kit, respectively, following the manufacturer’s instructions.

### Western blot analysis

Cells were lysed using RIPA buffer supplemented with aprotinin (Sigma), and Halt Protease Inhibitor Cocktail (100X) (Thermo Scientific). Protein samples were separated by sodium dodecyl sulfate–polyacrylamide gel electrophoresis (SDS-PAGE) and transferred to a nitrocellulose membrane (Bio-Rad, Mississauga, ON). Primary antibodies used in this study include caspase-1 (Sant Cruz Biotechnology, Dallas, TX), cleaved caspase-1 (AdipoGen Life Science, San Diego, CA), caspase-3 (Sant Cruz Biotechnology, Dallas, TX), cleaved caspase-3 (Cell signaling technology, Danvers, MA), HMGB1 (Cell signaling technology, Danvers, MA), MLKL (Abcam, Boston, MA), phosphorylated MLKL (Abcam, Boston, MA), CRT (Abcam, Boston, MA), and GAPDH (Sant Cruz Biotechnology, Dallas, TX). The expression levels Relative expression of proteins were visualized using the corresponding secondary HRP-conjugated antibodies and Amersham ECL select western blotting detection reagent, as described previously (46).

### TUNEL staining

TUNEL staining was conducted on 4T1 cells at 0.5, 1, 2 and 4 hours after 5-ALA-PDT according to the manufacturer’s protocol (Abcam; Cambridge, MA). Briefly, the cells were collected, resuspend in PBS and then fixed with 4% paraformaldehyde. The cells were labelled with Br-dUTP for 60 mins at 37°C, and then incubated in anti-BrdU-Red antibody for 30 mins. TUNEL positive cells were analyzed by flow cytometry using a BD FACSCalibur (BD Biosciences, San Jose, CA, USA).

### Transmission electron microscopy (TEM)

Cells were fixed in Karnovsky fixative (2% paraformaldehyde and 2.5% Glutaraldehyde in 0.1 M sodium cacodylate buffer (Sigma-Aldrich) for 20 mins, and post-fixed in 1% osmium tetroxide (Sigma-Aldrich) dissolved in 0.1 M sodium cacodylate (Sigma-Aldrich) buffer pH 7.4. Fixed cells were dehydrated in increasing concentrations (70–100%) of ethanol and acetone, and then embedded in BEEM resin capsules and polymerized overnight at 70°C. Ultrathin sections were cut at 80-100 nm and mounted on 300 mesh copper grids, stained with lead citrate (Electron Microscopy Sciences, Hatfield, PA), and observed using an FEI Tecnai G2 Spirit transmission electron microscope (FEI, Hillsboro, OR) operating at 80 KV.

### Animal studies

Female BALB/c mice obtained from Charles River Laboratories (Montreal, QC) were housed in a barrier unit within the central animal care facility in the Health Sciences Center at the Memorial University of Newfoundland. All animal experiments were conducted following the guidelines set by the Canadian Council on Animal Care and as approved by the Institutional Animal Care Committee of Memorial University Animal care committee. 4T1 cells were grown in DMEM with 10% FBS and antibiotics in a humidified atmosphere of 5% CO_2_ at 37°C. 5-ALA-PDT supernatant was collected from 4T1 cells treated with 5-ALA at the concentration of 50 μM for 4 hours prior to 1.5 mins of irradiation using a Theralase TLC 3000A modular light source. The supernatant of treated cells was collected at 24 hours after PDT treatment and centrifuged at 1300 rpm to remove the cell debris. For the freeze and thaw group, 4T1 cells were trypsinized and centrifuged to obtain cell pellets. Cells were resuspended in serum-free media and then subjected to three freeze and thaw cycles, followed by centrifugation at 1300 rpm to remove cell debris. At 8 weeks of age, female BALB/c mice randomly separated into 3 groups were injected with 30μl of cell-free media, supernatant of freeze/thaw cells, or supernatant of PDT cells intramuscular (*i.m.*) every week for 4 weeks. Resting for a week, at week 6, mouse mammary carcinoma 4T1 cells prepared in sterile normal saline (2x10^6^ cells/100μl) were injected into the right hind flank of BALB/c mice. We monitored the tumor growth for 12 days before the mice were sacrificed.

### Statistical analysis

Two-way ANOVA with *post-hoc* Tukey, Student’s t-test or Kruskal-Wallis test were performed using Prism (9.5.0) or IBM SPSS Statistics 27 as indicated in figure legends.

## Results

### Inflammatory cell death is responsible for PDT-induced cancer cell death

**Figure 2:**
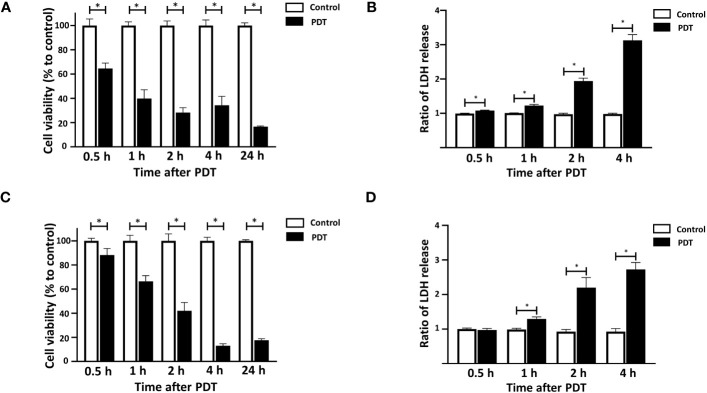
Time courses of 5-ALA-PDT induced cell death in 4T1 and DLD-1 cells. Mouse mammary carcinoma 4T1 cells **(A, B)** or human colon cancer DLD-1 cells **(C, D)** were treated with 5-ALA (50 μM for 4T1 and 250 μM for DLD-1) (PDT) or left untreated (Control) and for 4 hours, and then exposed to the light (618–630 nm/wavelength) for 1.5 mins. At the indicated time points after the light exposure, cell viability was measured using the CCK-8 assay kit **(A, C)** and LDH in culture was quantified using LDH cytotoxicity assay kit **(B, D)**. Mean ± SD relative cell viability and LDH release to control from 4 independent experiments. *p<0.01 by student’s t-test.

4T1 and DLD-1 cells were incubated with 5-ALA at concentrations of 50 μM and 250 μM for 4 hours followed by 1.5 mins light exposure, respectively. Cell viability was detected at 0.5, 1, 2, 4 and 24 hours after light treatment and LDH release was analyzed at 0.5, 1, 2 and 4 hours after light treatment for 4T1 cells and DLD-1 cells. Distinct 5-ALA-PDT conditions were used for 4T1 and DLD-1 cells as their sensitivities to 5-ALA-PDT were different. 5-ALA-PDT significantly reduced cell viability and increased LDH levels in the supernatant at 0.5, 1, 2 and 4 hours after light exposure in 4T1 cells (**Figures 2A, B**). Cell death was similarly induced from 0.5 hour after light exposure in DLD-1 cells (**Figures 2C, D**).

**Figure 3:**
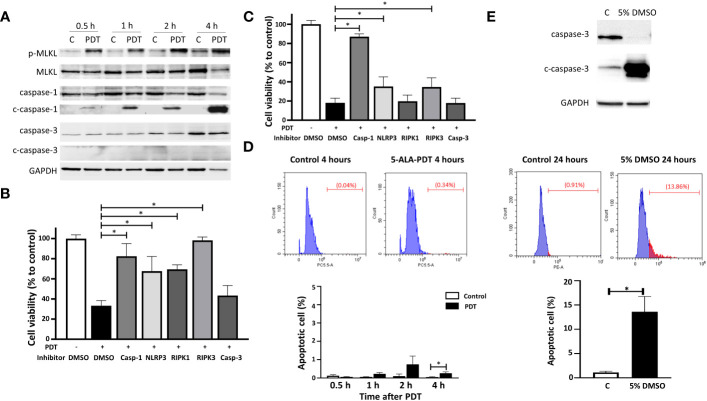
5-ALA-PDT causes cancer cell death via necroptosis and pyroptosis, but not apoptosis. **(A)** 4T1 cells were treated with 5-ALA (50 μM) (PDT) or left untreated (C) for 4 hours, and then exposed to light (618–630 nm/wavelength) for 1.5 mins. At 0.5, 1, 2, and 4 hours after light exposure, cell lysates were collected for western blot analysis using antibodies against phosphorylated MLKL (p-MLKL), MLKL, caspase-1, cleaved caspase-1 (c-caspase-1), caspase-3, cleaved caspase-3 (c-caspase-3) and GAPDH. **(B)** 4T1 or **(C)** DLD-1 cells were treated with vehicle control (DMSO), caspase-1 inhibitor (Z-WEHD-FMK, 20 μM), NLRP inhibitor (MCC950, 20 μM), RIPK1 inhibitor (Nec-1s, 20 μM), RIPK3 inhibitor (Dabrafenib, 10 μM) or caspase-3 inhibitor (Z-DEVD-FMK, 20 μM) in combination with 5-ALA treatment (50 μM (4T1) and 250 μM (DLD-1)) (PDT +) or left untreated (PDT -) for 4 hours. Cells were then exposed to light (618–630 nm/wave) for 1.5 mins. At 4 hours post light exposure, the cell viability was measured using the CCK-8 assay kit. Mean ± SD relative cell viability to control from 4 independent experiments. *p<0.01 by Two-way ANOVA. **(D)** 4T1 cells treated with or without 5-ALA-PDT were subjected to TUNEL staining followed by flow cytometry analysis. Mean ± SD from 3 independent experiments. *p<0.01 by the student’s t test. **(E)** Apoptotic 4T1 cells treated with or without 5% DMSO for 24 hours were determined by western blot analysis using antibodies against caspase-3, cleaved caspase-3 and GAPDH, and by TUNEL staining followed by flow cytometry analysis. Mean ± SD from 3 independent experiments. *p<0.01 by the student’s t test.

To clarify if 5-ALA-PDT activates programmed cell death (PCD) pathways, we determined the expression of PCD markers such as phosphorylated MLKL (necroptosis), cleaved caspase-1 (pyroptosis) and cleaved caspase-3 (apoptosis) in 4T1 cells treated with or without 5-ALA-PDT (50 μM 5-ALA for 4 hours, 1.5 mins of light exposure). MLKL was phosphorylated at 0.5, 1, 2 and 4 hours after light exposure. 5-ALA-PDT decreased caspase-1 but increased cleaved caspase-1, indicating that caspase-1 is activated. In contrast, the apoptosis pathway was not activated as the amount of caspase-3 and cleaved caspase-3 did not show any difference between control cells and those treated with 5-ALA-PDT (**Figure 3A** and **Supplementary Figure 1**).

To determine if necroptosis and/or pyroptosis are responsible for cell death induced by 5-ALA-PDT, 5-ALA-PDT was conducted on 4T1 or DLD-1 cells in the presence or absence of the PCD inhibitors. 34% of 4T1 cells treated with vehicle control (DMSO) survived at 4 hours after 5-ALA-PDT. Treatment with caspase-1 (pyroptosis), NLRP3 (pyroptosis), RIPK1 (necroptosis) or RIPK3 (necroptosis) inhibitor significantly reduced cell death induced by 5-ALA-PDT while caspase-3 inhibitor (apoptosis) did not increase cell viability of 5-ALA-PDT treated 4T1 cells (**Figure 3B**). These results demonstrate that necroptosis (RIPK1 and RIPK3 dependent) and pyroptosis, but not apoptosis, are responsible for 5-ALA-PDT induced cell death in 4T1 cells. On the other hand, cell death induced by 5-ALA-PDT was reduced in DLD-1 cells treated with caspase-1, NLRP3 or RIPK3 inhibitors, while caspase-3 or PIPK1 inhibitor did not influence the cell death (**Figure 3C**), suggesting that necroptosis (RIPK3 dependent) and pyroptosis are responsible for 5-ALA-PDT induced cell death in DLD-1 cells. These results agree with the previous study demonstrating RIPK-dependent cell death in glioblastoma (47). To further determine apoptosis induction in 4T1 cells treated with 5-ALA-PDT, TUNEL staining followed by flow cytometry analysis was conducted (**Figure 3D**). We found that TUNEL positive cells induced by 5-ALA-PDT were less than 2% throughout the experiment while apoptotic cells were significantly more in 4T1 cells at 4 hours after 5-ALA-PDT than in control 4T1 cells. Moreover, we confirmed that apoptosis induction in 4T1 cells treated with 5% DMSO was detected by western blot analysis using cleaved caspase-3 antibody and TUNEL staining (**Figure 3E**), demonstrating that our experimental system can detect apoptotic cells efficiently.

**Figure 4:**
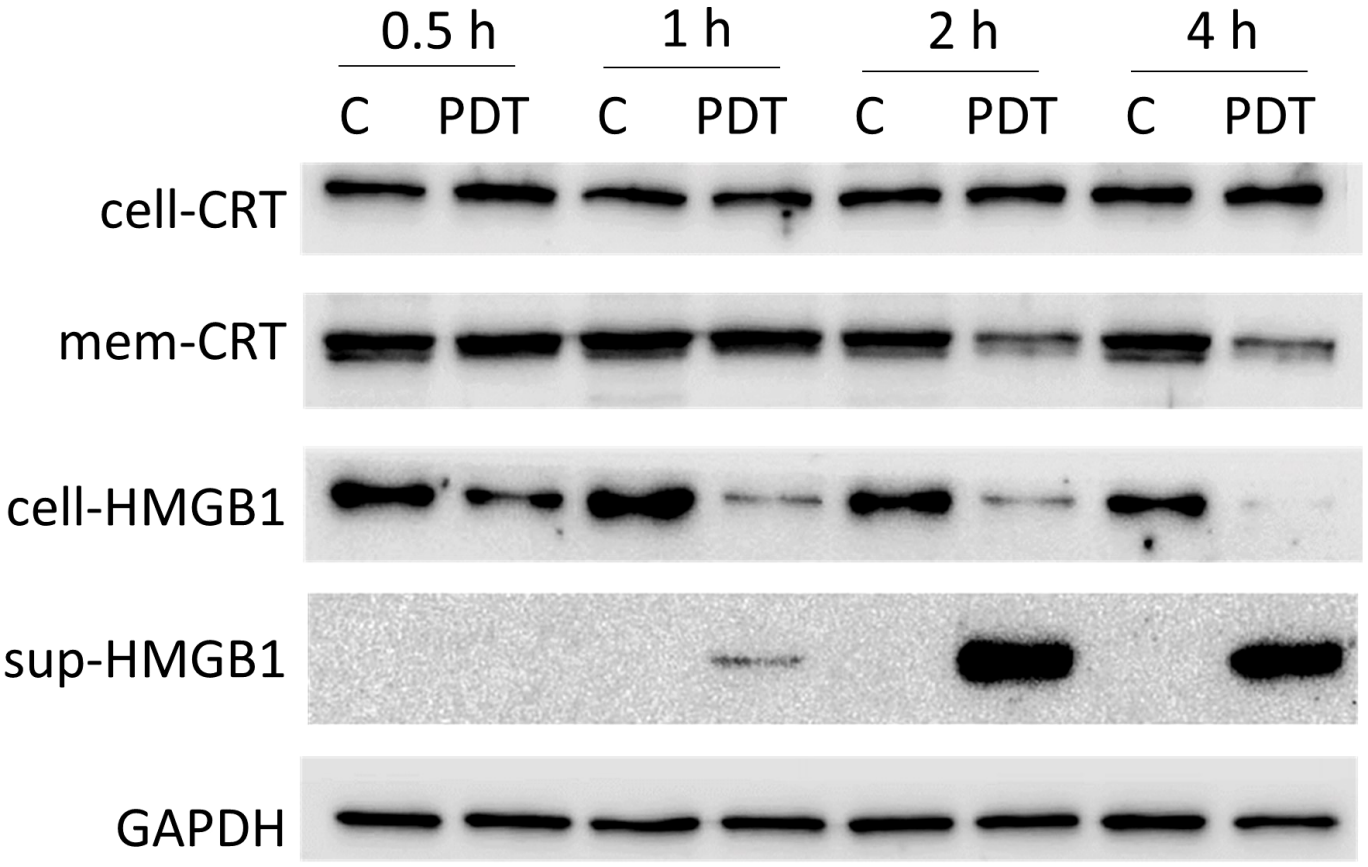
Expression of CRT and HMGB1 in 4T1 cells treated with 5-ALA-PDT. 4T1 cells were treated with 5-ALA (50 μM) (PDT) or left untreated (C) for 4 hours, and then exposed to light (618–630 nm/wavelength) for 1.5 mins. At 0.5, 1, 2, and 4 hours after light exposure, proteins from the cytoplasm, from the cellular membrane and in the supernatants were collected for western blot analysis using antibodies against CRT (whole cell CRT, cell-CRT; membrane CRT, mem-CRT), HMGB1 (whole cell HMGB1, cell-HMGB1; supernatant HMGB1, sup-HMGB1) and GAPDH.

Accumulation of CRT in the cellular membrane is observed in apoptotic or pre-apoptotic cells (48). HMGB1 is one of the inflammatory mediators released during inflammatory cell death (49). To determine whether these changes of CRT and HMGB1 were induced in 4T1 cells treated with 5-ALA-PDT, we conducted western blot analysis of CRT in the cellular cytoplasmic and membrane fractions, and of HMGB1 in the cells and the supernatants (**Figure 4** and **Supplementary Figure 2**). As results, we did not observe the translocation of CRT from the cytoplasm to the membrane caused by 5-ALA-PDT. In contrast, HMGB1 expression was decreased in the cells and increased in the supernatant from 1 hour after 5-ALA-PDT, indicating that inflammatory cell death is induced.

**Figure 5:**
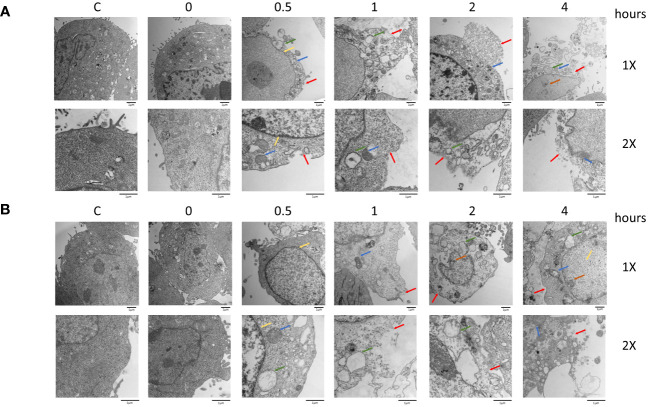
Morphological changes after PDT in 4T1 and DLD-1 cells. Mouse mammary carcinoma 4T1 cells **(A)** or human colon cancer DLD-1 cells **(B)** were treated with 5-ALA (50 μM for 4T1 and 250 μM for DLD-1) (PDT) or left untreated (Control) for 4 hours, and then exposed to light (618–630 nm/wavelength) for 1.5 mins. At the indicated time points after 5-ALA-PDT, morphological changes were investigated by TEM analysis. Plasma membrane permeabilization (red arrow), organelle swelling and vacuolization (green arrow), mitochondrial swelling (blue arrow), swollen nucleus (yellow arrow), and deficiency of nuclear chromatin (orange arrow) are indicated.

Morphological changes induced by 5-ALA-PDT were examined in 4T1 and DLD-1 cells by transmission electron microscopy (TEM) (**Figure 5**). In both cell lines, we observed typical morphological features of pyroptosis and necroptosis such as plasma membrane permeabilization (red arrow), organelle swelling and vacuolization (green arrow), mitochondrial swelling (blue arrow) and deficiency of nuclear chromatin (yellow arrow) (50, 51). In contrast, morphological features of apoptosis such as chromatin condensation and relocation were not found in the TEM analysis. The results in **Figures 3**, **5** clearly suggest that 5-ALA-PDT induces pyroptosis and necroptosis but not apoptosis.

### 5-ALA-PDT-induced cell death initiates anticancer immunity

**Figure 6:**
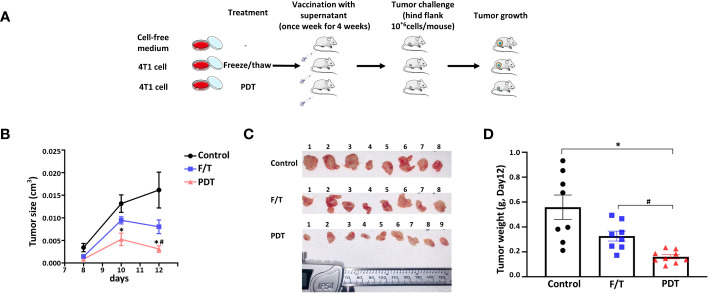
5-ALA-PDT stimulates antitumor immunity in mice. **(A)** BALB/c mice were injected intramuscular (*i.m.*) with 30 μl of cell-free media (Control), supernatant of freeze/thaw 4T1 cells (FT) or supernatant of 5-ALA-PDT treated 4T1 cells (PDT) every week for 4 weeks (n=8-9/group). Resting for a week, at week 6, 4T1 cells were injected (2x10^6^ cells/100 μL) into the right hind flank of the mice. **(B)** Tumor measurement at 8, 10 and 12 days after 4T1 cell injection, **(C)** images of the tumors removed from mice at 12 days after 4T1 cell injection and **(D)** tumor weight at 12 days after 4T1 cell injection. *p<0.05 and # *p*<0.05 by Two-way ANOVA, * compared with control group, # compared with F/Z group **(B)** and *p<0.05 by Kruskal-Wallis test **(D)**.

Next, we determined whether the inflammatory cell death initiated by 5-ALA-PDT leads to the induction of anticancer immunity *in vivo*. We injected female 8-weeks-old BALB/c mice *i.m.* with serum-free medium (Control), freeze and thaw 4T1 supernatant (F/T) or 5-ALA-PDT 4T1 supernatant (PDT) once a week for 4 weeks. Resting for a week, we then challenged mice with *s.c.* injections of 4T1 cells at the right hind flank (**Figure 6A**). Tumor growth in mice treated with PDT supernatant was inhibited compared to those treated with the cell-free medium at 10 and 12 days after 4T1 cell injection (**Figure 6B**). In contrast, tumor growth was significantly inhibited in mice treated with the F/T supernatant only 12 days after 4T1 cells injection. The result of tumor measurements was confirmed visually by images of tumors removed from the mice 12 days after 4T1 cell injection (**Figure 6C**). Moreover, we found that the weight of tumors from mice treated with PDT supernatant were significantly smaller than those from mice treated with F/T supernatant or cell free medium, while the F/T supernatant treatment did not reduce the tumor weight significantly (**Figure 6D**). These results demonstrate that cell death caused by 5-ALA-PDT initiates anticancer immunity in an animal model of cancer.

## Discussion

The immunosuppressive microenvironment of tumors is one of the major obstacles for cancer therapies. Standard cancer therapies, such as chemotherapy and radiotherapy, could cause inflammatory responses and break the immune silence of tumors, which may further promote therapeutic efficacies in cancer patients and lead to cancer cure (40, 52, 53). Induction of inflammatory cell death by the cancer therapies is the first essential step to establish systemic anticancer immunity. The objective of the current study was to investigate whether 5-ALA-PDT could initiate anticancer immunity by inducing inflammatory cell death of cancer cells. We found that 5-ALA-PDT causes cancer cell death by inducing pyroptosis and necroptosis, which are a form of inflammatory cell death. Moreover, the supernatant of cells treated with 5-ALA-PDT was effective to induce anticancer immunity *in vivo*. These results demonstrate that 5-ALA-PDT can kill cancer cells in an immunogenic manner that initiates anticancer immunity, which sheds light on a new therapeutic aspect of 5-ALA-PDT.

We used a lower amount of 5-ALA in this study than those used in our previous studies which focused on the efficacy of direct cancer cell killing by 5-ALA-PDT (5). In fact, 5-ALA-PDT does not induce inflammatory cell death when higher concentrations of 5-ALA were used (data not shown), suggesting that cancer cells undergo other types of cell death, such as apoptosis and necrosis. This finding agrees with a previous study reported by M Korbelik and his colleagues that the cancer vaccine generated by chlorin e6-based PDT was more effective to reduce mouse SCCVII squamous cell carcinoma cells when lower concentrations of the photosensitizer were used (54). It is unknown how the degree of cell damage could influence the induction of different types of cancer cell death. Nevertheless, it should be noted that 5-ALA-PDT conditions could be optimized independently for induction of cell lysis (direct cancer killing) and of anticancer immunity (breaking immune silence) in clinical settings.

Previous studies demonstrated that 5-ALA-PDT causes cancer cell death by inducing apoptosis (17, 44, 55–59). However, we did not find the expression of the apoptosis marker (cleaved caspase-3) or positive TUNEL staining in 5-ALA-PDT treated cells (**Figures 3A, D**) or the reduction of 5-ALA-PDT-induced cell death by the apoptosis inhibitor (**Figures 3B, C**). Moreover, RIPK1 was involved in 5-ALA-PDT-induced cell death of 4T1 cells but not that of DLD-1 cells (**Figures 3B, C**). We believe that these discrepancies may be caused by differences in potency of 5-ALA-PDT or cancer cell lines used in the studies. As mentioned above, high dosage of 5-ALA-PDT could lead to non-inflammatory cell death such as apoptosis and necrosis while low doses of 5-ALA-PDT, which kills cancer cell slowly, may be the key to induce inflammatory cell death. It is also known that the induction of the PCD is dependent on cell lines, which might lead to the differences between 4T1 and DLD-1 cells (60, 61). Furthermore, caspase-1 inhibitor increased cell viability more than NLRP3 inhibitor in 5-ALA-PDT treated DLD-1 cells (**Figure 3C**), suggesting that other upstream elements of caspase-1 may initiate pyroptosis in DLD-1 cells. More detailed studies are required to identify precise cellular signaling pathways of PCD activated by 5-ALA-PDT.

Supernatant from PDT treated cells was more effective to inhibit 4T1 tumor growth than the F/T supernatant, suggesting that the PDT supernatant contains components that initiate anticancer immunity (**Figure 6**). Oxidative stress induced by 5-ALA-PDT could generate oxidation-associated molecular patterns, which may increase the immunogenicity of 4T1 tumor cells (3, 62). Furthermore, cytokines and inflammatory mediators in PDT supernatant, which are produced during pyroptosis or necroptosis, could also promote antitumor immunity in the presence of the tumor antigens. Cytokines and inflammatory mediators induced by pyroptosis, or necroptosis include IL-1β, IL-6, IL-18, TNF-α, DAMPs and PAMPs. It is of interest to identify which of these cytokines and inflammatory mediators play critical roles in initiating anticancer immunity following 5-ALA-PDT.

## Data availability statement

The raw data supporting the conclusions of this article will be made available by the authors, without undue reservation.

## Ethics statement

The animal study was approved by Institutional Animal Care Committee. The study was conducted in accordance with the local legislation and institutional requirements.

## Author contributions

The authors confirm their contribution to the paper as follows: study conception and design: KH and LW; data collection: LW, VC, NN, and ST; analysis and interpretation of results: LW and KH; draft manuscript preparation: LW and KH. All authors contributed to the article and approved the submitted version.

## Supplementary material

Supplementary Figure 1 Original gel images of **Figure 3A**. **(A)** phosphorylated-MLKL, **(B)** total MLKL, **(C)** total caspapse-1, **(D)** cleaved caspase-1, **(E)** total caspase-3 and **(F)** GAPDH.

Supplementary Figure 2 Original gel images of **Figure 4**. **(A)** whole cell CRT, **(B)**membrane CRT, **(C)**whole cell HMGB1, **(D)** supernatant HMGB1 and **(E)** GAPDH.
