# Supplementary figures and images for: Inflammatory cell death induced by 5-aminolevulinic acid-photodynamic therapy initiates anticancer immunity
Source: Front Oncol. 2023 Oct 2;13:1156763. doi: 10.3389/fonc.2023.1156763 (PMC10581343; doi:10.3389/fonc.2023.1156763)

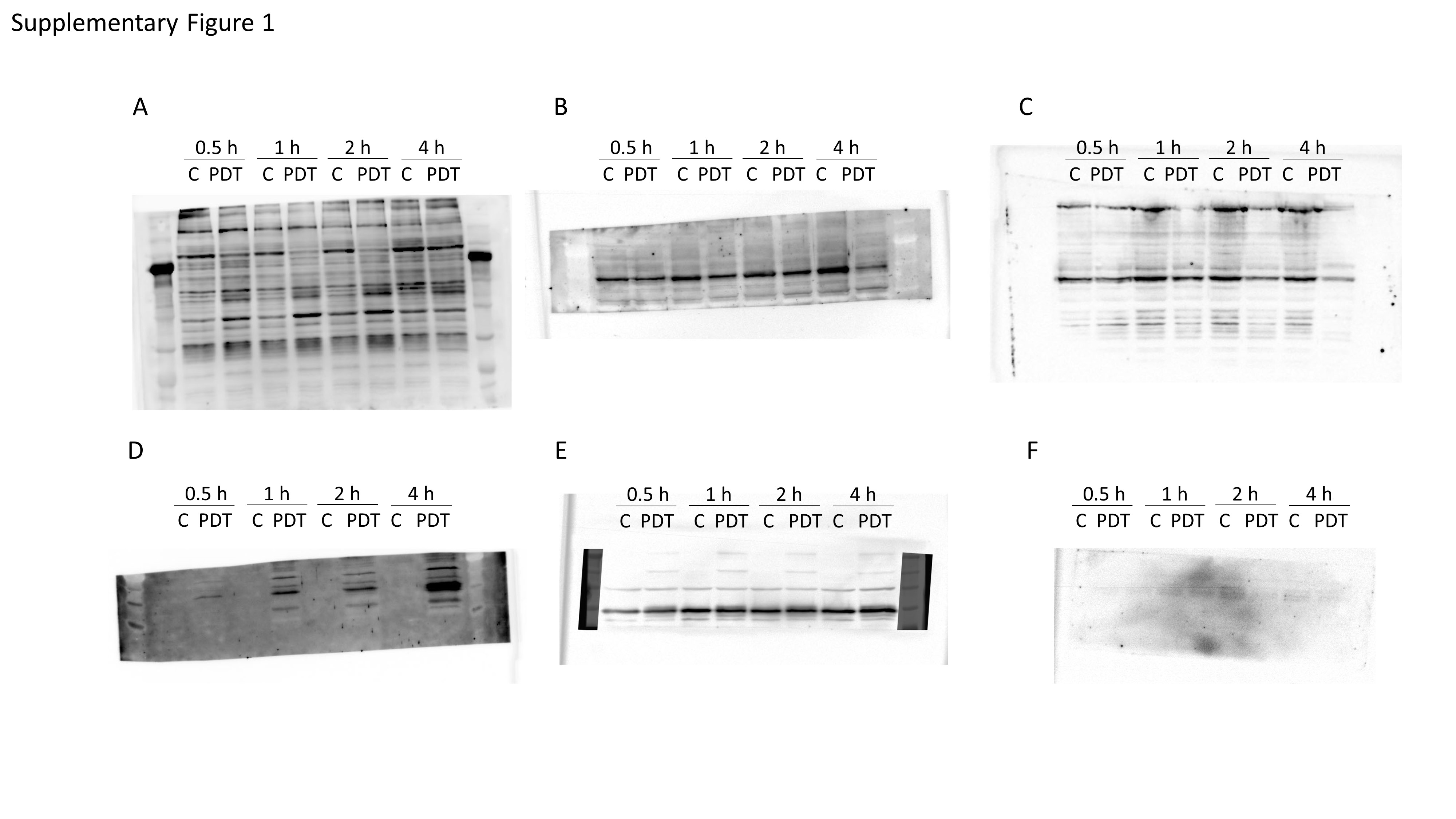

Supplement: Supplementary Figure 1 — Original gel images of Figure 3A . (A) phosphorylated-MLKL, (B) total MLKL, (C) total caspapse-1, (D) cleaved caspase-1, (E) total caspase-3 and (F) GAPDH. [file Image_1.jpeg]

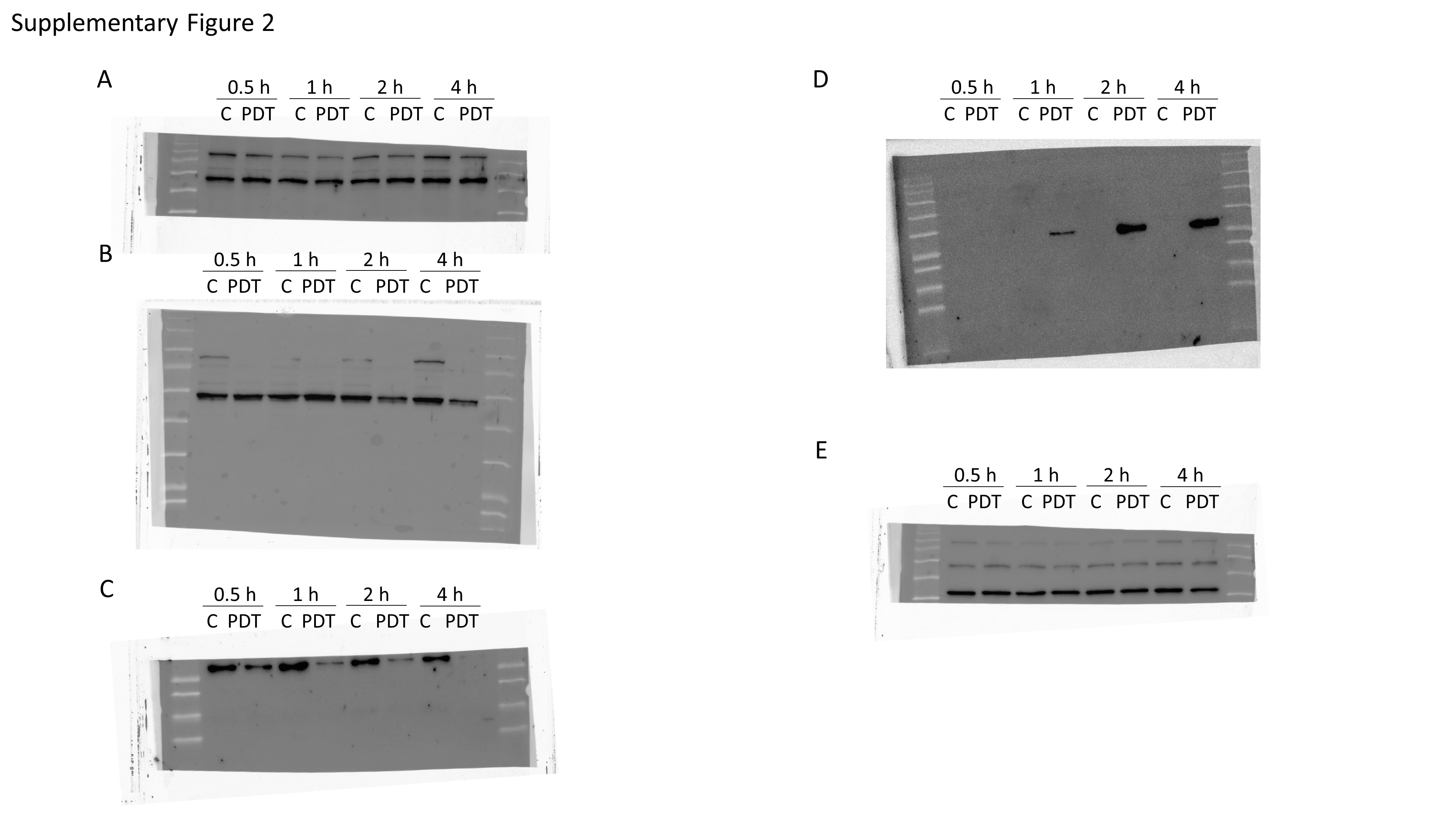

Supplement: Supplementary Figure 2 — Original gel images of Figure 4 . (A) whole cell CRT, (B)membrane CRT, (C)whole cell HMGB1, (D) supernatant HMGB1 and (E) GAPDH. [file Image_2.jpeg]
